# FACS-Seq analysis of *Pax3*-derived cells identifies non-myogenic lineages in the embryonic forelimb

**DOI:** 10.1038/s41598-018-25998-1

**Published:** 2018-05-16

**Authors:** Arun J. Singh, Chih-Ning Chang, Hsiao-Yen Ma, Stephen A. Ramsey, Theresa M. Filtz, Chrissa Kioussi

**Affiliations:** 1 Department of Pharmaceutical Sciences, College of Pharmacy, Oregon State University, Corvallis, Oregon, 97331 USA; 2 Molecular Cell Biology Graduate Program, Oregon State University, Corvallis, Oregon, 97331 USA; 3 Department of Biomedical Sciences, College of Veterinary Medicine, Oregon State University, Corvallis, Oregon, 97331 USA; 4 School of Electrical Engineering and Computer Science, Oregon State University, Corvallis, Oregon, 97331 USA

## Abstract

Skeletal muscle in the forelimb develops during embryonic and fetal development and perinatally. While much is known regarding the molecules involved in forelimb myogenesis, little is known about the specific mechanisms and interactions. Migrating skeletal muscle precursor cells express Pax3 as they migrate into the forelimb from the dermomyotome. To compare gene expression profiles of the same cell population over time, we isolated lineage-traced *Pax3*^+^ cells (*Pax3*^*EGFP*^) from forelimbs at different embryonic days. We performed whole transcriptome profiling via RNA-Seq of *Pax3*^+^ cells to construct gene networks involved in different stages of embryonic and fetal development. With this, we identified genes involved in the skeletal, muscular, vascular, nervous and immune systems. Expression of genes related to the immune, skeletal and vascular systems showed prominent increases over time, suggesting a non-skeletal myogenic context of *Pax3*-derived cells. Using co-expression analysis, we observed an immune-related gene subnetwork active during fetal myogenesis, further implying that *Pax3*-derived cells are not a strictly myogenic lineage, and are involved in patterning and three-dimensional formation of the forelimb through multiple systems.

## Introduction

Skeletal muscle formation in the forelimb during embryogenesis is a tightly regulated and controlled process. Forelimb muscles derive from paraxial mesoderm-derived anatomical structures called somites. Somites segment themselves into the myotome, sclerotome, and dermomyotome. The dermomyotome is divided into epaxial and hypaxial layers, the latter of which is the origin of all skeletal muscle of the trunk and back^1,2^. Pax3 is a homeodomain sequence-specific transcription factor (SSTF) that marks all somite-derived skeletal muscles in the forelimb. Pax3 is expressed starting at embryonic day (E) 10 in embryonic myogenic progenitor cells (EMPCs), which triggers migration and delamination of EMPCs from the ventrolateral lip of the hypaxial dermomyotome into the limb bud^3–6^. In *Pax3* knockout (KO) mutant mice, myogenic progenitor cells fail to migrate and delaminate from the somite, which ultimately leads to a forelimb deficient of skeletal muscle^7,8^.

After EMPCs colonize the limb bud, skeletal muscle forms in distinct, successive stages^9^. Between E10 and E12, embryonic myoblasts fuse into embryonic myotubes. Between E12 and E16, fetal myoblasts fuse with both each other and embryonic myotubes to form fetal myofibers that serve as the foundation for future skeletal muscle. During this process, significant changes occur in gene expression^10^ and the underlying gene regulatory networks^11,12^, but little information is known regarding specifics that drive the molecular processes. Many of the mechanisms that take place during myogenesis are re-activated during skeletal muscle regeneration in adults, including the activation of skeletal muscle-specific SSTFs^13^, making it possible to translate any insights gained between systems. Since all known forelimb skeletal muscles derive from Pax3^+^ progenitor cells, the *Pax3*^*EGFP*^ lineage offers a genetic tool to uncover the molecular processes that determine forelimb myogenesis and organogenesis. By observing the gene expression profiles of *Pax3*^*EGFP*^ cells across the developmental time course as they migrate from the dermomyotome into forelimb, we can identify the molecular players coincident with muscle stages as they are formed and maintained in coordination with other cell lineages in the developing limb structure.

Network analysis is a quantitative paradigm for analyzing biological systems as individual parts working and interacting together^14–16^. Technological advances combined with reduced prices in next-generation sequencing have resulted in development of advanced techniques for network analysis of cell specific changes in organ development and disease^17^. Graphical representation via network analysis of gene expression data enables the visualization of complex interactions in large data sets in an intuitive format. In such a representation, nodes represent genes that are then connected to each other via edges that represent interactions. A specific type of network, co-expression networks, are created from transcriptomics data to reveal patterns of gene expression in dynamic systems^18–20^, and have been used to identify cell-type specific patterns of gene expression during development and the changes in regulatory interactions responsible for cell-state phenotypes^21,22^, among other uses.

Applying co-expression analysis to *Pax3*^*EGFP*^ lineage-traced myoblasts provides a model system to decode the mechanisms behind embryonic and fetal myogenesis in the forelimb. In this study, we used next generation RNA sequencing of lineage-traced cells isolated through fluorescent-activated cell sorting (FACS-Seq) to perform differential expression and co-expression analysis during distinct stages of embryonic development. We discovered that the *Pax3*^*EGFP*^ lineage harbors several cell populations not previously defined, including cells that will likely populate the immune and hematopoietic systems parallel to the already known skeletal muscle, smooth muscle, and neuronal systems. Development of these diverse systems is tightly orchestrated as cells migrate from the dermomyotome, enter the forelimb space, and receive signals from the highly plastic environment. SSTFs integrate external signals during patterning with shifting gene expression networks that coordinate the migration, proliferation, differentiation, and integration of cell types into fully functioning organs and multi-system limb structures. For example, homeodomain SSTFs in combination of *Shh*, *Fgf* and *Wnt* signaling dominate the early patterning events in embryonic forelimb myogenesis, followed by the rise in importance of zinc-finger and helix-turn-helix SSTFs in fetal states. In this study, we observed that *Pax3*-derived cells contribute more fully to the three-dimensional formation of the forelimb than previously thought, and give rise to cells with characteristics of the skeletal, vascular, nervous, hemolymphoid and immune systems in addition to muscle. Thus, the dermomyotome might give rise to more many cell populations than originally thought.

## Results and Discussion

### Isolation of *Pax3-*derived embryonic forelimb cells

**Figure 1:**
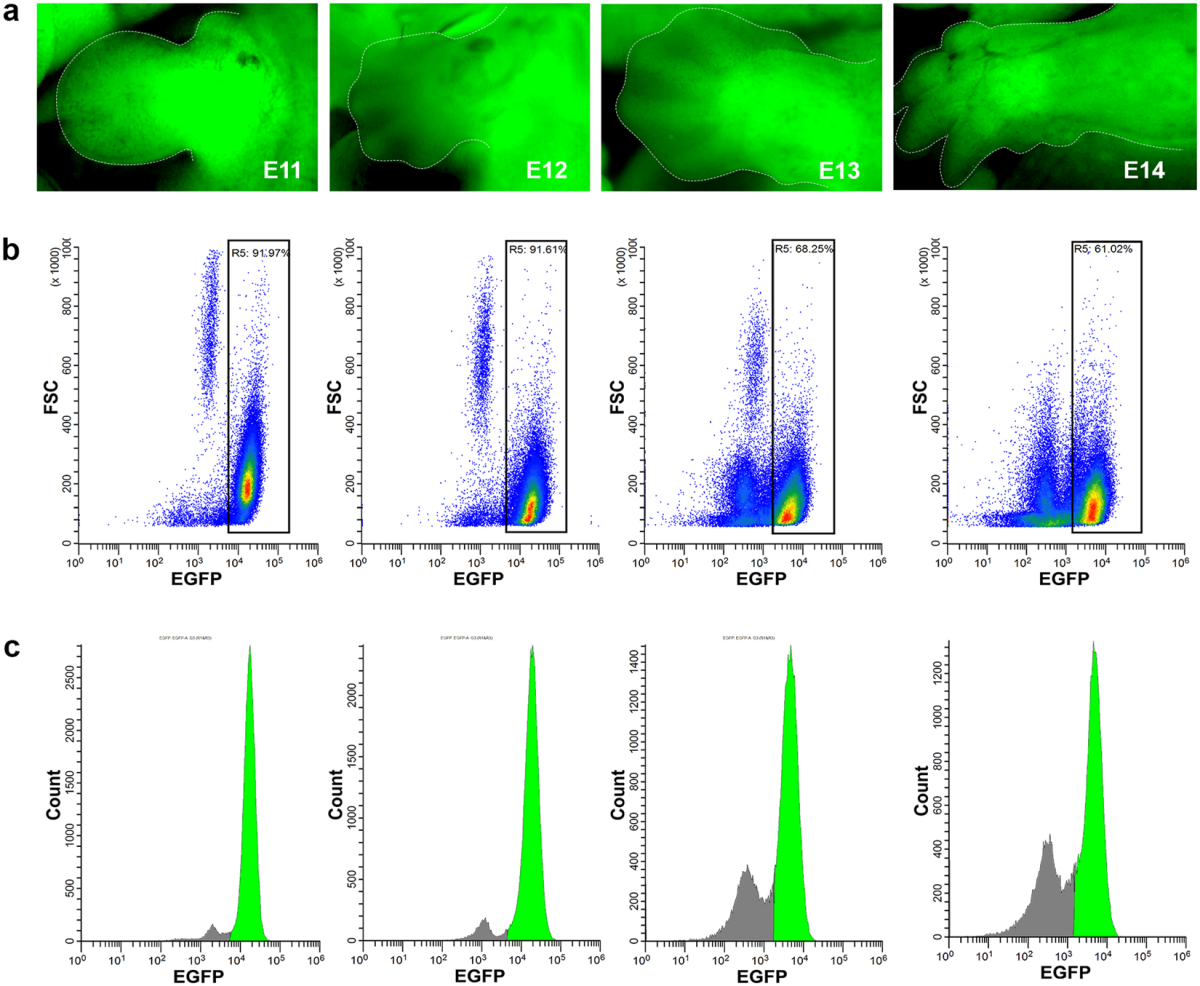
*Pax3*^*EGFP*^ expression in mouse embryonic forelimbs. **(a)** Fluorescent microscopy showing Pax3^EGFP^ expression based on a Pax3^Cre^|Rosa26^EGFP^ driver at E11, E12,  E13, and E14 forelimbs. **(b)** Scatter plots from FACS showing EGFP intensity on the x-axis, and forward scatter (FSC) on the y-axis. Gate R5 shows 92%, 92%, 68%, and 61% EFGP-positive cells in forelimbs at E11, E12, E13, and E14, respectively. **(c)** Histograms depict EGFP intensity on the x-axis vs cell number (count) on the y-axis. Green peaks represent EFGP-positive populations based on gating from R5.

To trace the genes involved in myogenesis in the forelimb in real time, we used a transgenic mouse model genetically composed of a *Pax3*^*Cre*^ driver^23^ combined with a *ROSA26*^*EGFP*^ tracer^24^. When both genotypes are combined into one mouse, all cells that at any point ever expressed Pax3 will also express EGFP, including any and all daughter cells (lineage tracer). This system enables the tracking of the same cell population in the mouse forelimb over time as it develops and differentiates. We chose E11, E12, E13, and E14 as time points for analysis to trace development from the beginning of embryonic myogenesis, when the Pax3^+^ dermomyotome-derived cells enter the myogenic lineage, to the onset of fetal myogenesis, when the myoblasts/myotubes start to form myofibers. Mouse embryos at each stage show strong EGFP expression, especially in the forelimbs (Fig. 1a). As the forelimb develops, individual digits and muscle groups develop too, seen clearly at E14. FACS^25^ was used to isolate EGFP expressing cells (*Pax3*^*EGFP*^) at each stage. Density-based scatter plots that represent EGFP fluorescence intensity vs. cell size show two distinct cell populations in each stage (Fig. 1b), EGFP-positive and EGFP-negative cells. A histogram representation gives a more clear image of the two distinct cell populations (Fig. 1c). *Pax3*^*EGFP*^ cells comprise 92% of the whole cell population of the forelimb at E11 and E12 (Fig. 1b) in agreement with strong EGFP-fluorescence seen by microscopy (Fig. 1a). At E13, the *Pax3*^*EGFP*^ cell population was reduced to 68% (and was further reduced at E14) due to reduced efficiency of our tissue disaggregation/cellular dissociation procedure (Fig. 1a,b). The onset of fetal myogenesis occurs between E12 and E13, when embryonic myofibers fuse with fetal myoblasts/myotubes to form fetal myofibers. The cytoskeletal rearrangements that occur among cells at E12-E13 generates a larger extracellular matrix which imparts resistance to our enzo-mechanical dissociation process (see Materials and Methods), and many cells were filtered out as clumps including dense tissue that failed to dissociate. The exact genes and molecular mechanisms involved in this process remain elusive and would be interesting to study.

### Gene expression profiling of *Pax3-*derived embryonic forelimb cells

**Figure 2:**
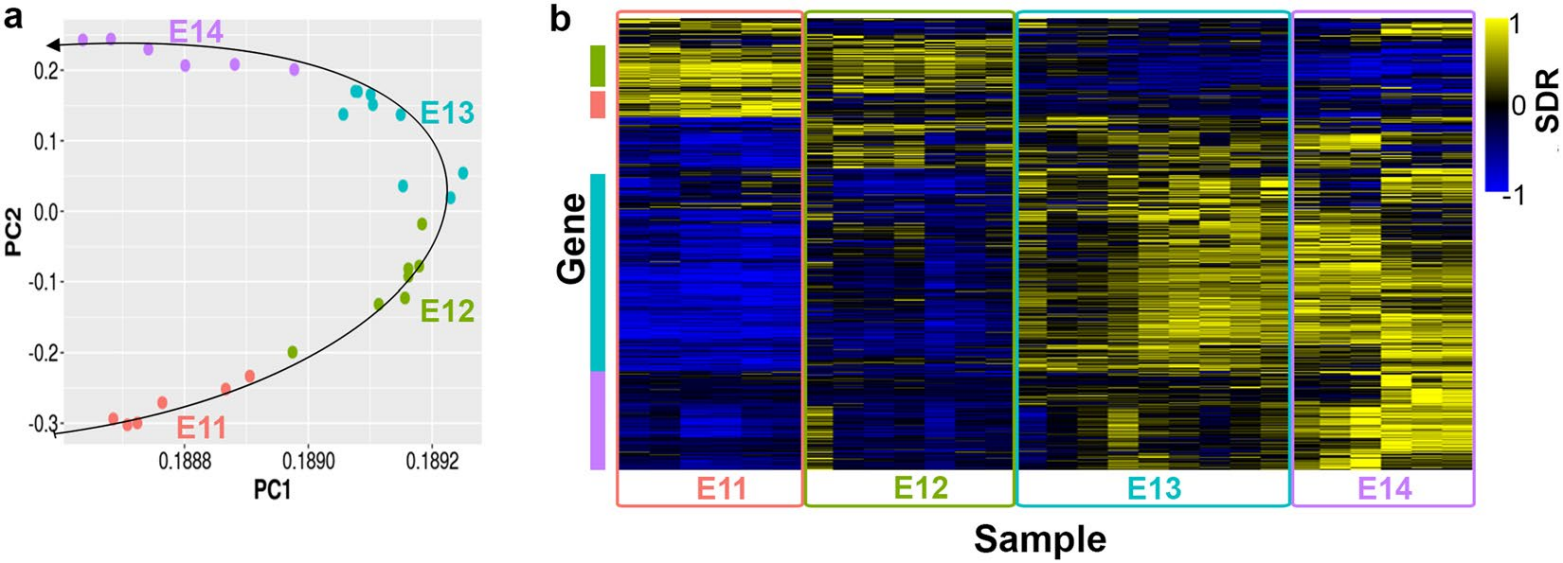
Differential expression (DE) and Gene Ontology (GO) term analysis of RNA-Seq data from sorted, *Pax3*^*EGFP*^ cells. **(a)** Principal component analysis (PCA) and plot of all 28 samples. PCA shows good clustering of samples by biological time point, with variation between samples in the same group. Samples appear to follow the developmental trajectory. **(b)** Heatmap of signed difference ratio (SDR) based on all 4,481 DE genes between any two consecutive developmental states. Columns represent samples, and each row represents one DE gene. Yellow indicates high expression and blue indicates low expression, relative to the average expression of each gene between all samples. Red, green, blue, and purple bars on the left indicate clusters of DE genes expressed at E11, E11, E12, E13 and E14, respectively.

After sorting, total RNA from each sample was extracted and tested by the Bioanalyzer for quality control. Only high-quality samples with an RNA Integrity Number (RIN) above 7.0 were retained for library preparation and processed for sequencing. Upon aligning the mapped sequence reads to the published murine mm10 genome and calculating the differentially expressed (DE) genes between each time point, a quality control step was performed via principal component analysis (PCA)^26^. The PCA plot (Fig. 2a) shows the variability between biological replicates in our system, and emphasizes the value of ample biological replicates for a study like this. The PCA plot also shows a distinct clustering of samples by stage with greater biological variation between stages that among samples from the same stage. Interestingly, the clustering of samples by stages appears to follow a developmental trajectory. Samples from E11 cluster in the bottom left and follow a horizontal parabola-like trajectory through E14, suggesting that time of conception is a significant factor in our analysis, as would be expected. Vaginal plug checking to determine timing of pregnancy was performed only once per day and therefore litters could be up to 12 hours apart in age but still marked as the same embryonic day for analysis. Variability may be accentuated at early stages when developmental changes are more dramatic.

Gene expression levels were calculated for each sample. DE genes were determined and a heatmap was generated based on the signed difference ratio (SDR) from log_2_-normalized reads (Fig. 2b). Distinct clusters of genes form at each stage based on their expression patterns. Clusters of interest are graphically delineated with red, green, blue, and purple boxes to the left of the heatmap.

Genes segregated with the red cluster were expressed specifically at E11, implying that they are early embryonic myogenesis markers. Gene ontology (GO) term enrichment analysis of the red cluster revealed an overrepresentation of genes associated with pattern specification processes (false discovery rate, FDR = 6.49 × 10^−5^), neuron differentiation (FDR = 0.008), and appendage morphogenesis (FDR = 2.03 × 10^−4^). Example genes in these categories were primarily homeodomain SSTFs represented by the Hox family, in agreement with previous reports that associated the Hox genes with regulation of patterning and digit formation in the embryonic limb^27–29^, including the Hoxc and Hoxd family genes expressed at E11^10^.

Genes segregated within the green cluster were expressed during E11 and E12 (Fig. 2b), and marked embryonic myogenesis. GO term functional annotation enrichment analysis indicated that “green” genes are over-represented with those involved in epithelial tube morphogenesis (FDR = 0.009), central nervous system (CNS) development (FDR = 2.24 × 10^−4^), mesenchyme development (FDR = 9.66 × 10^−2^), and neuron fate commitment (FDR = 4.71 × 10^−4^), among others, suggesting that formation of the CNS is taking place during E11–E12. Since *Pax3*^*EGFP*^ cells are known to mark all cells in the skeletal muscle lineage in the forelimb, detection of many so genes that are not usually expressed in myoblasts was surprising.

Genes clustered in the blue box (Fig. 2b; Fig. S1) showed high expression levels at E13 and E14 which coincides with the onset of fetal development, and they were involved in angiogenesis (FDR = 2.61 × 10^−13^), negative regulation of cell proliferation (FDR = 2.46 × 10^−8^), and differentiation (FDR = 1.60 × 10^−8^). Example genes in the “blue” cluster included the angiogenesis markers *Angpt2* and *Anpep*, and negative markers of cell proliferation such as *Ar* and *Dpt*. Their expression suggests that in fetal states cells of the *Pax3*^*EGFP*^ lineage stop proliferating, exit the cell cycle and possibly enter the smooth muscle cell lineage. Angiogenesis and myogenesis are highly interrelated and co-dependent during forelimb development. Expression of certain angiogenesis-related genes can increase the rate of muscle regeneration in adult skeletal muscle^30,31^. Additionally, colonization of vascular cells in the developing forelimb is required for migration of Pax3^+^ myoblasts into the limb bud^32^, implying communication between muscle and vascular systems during development.

Genes in the purple cluster (Fig. 2b; Fig. S1) were expressed explicitly at E14 and were associated with immune response regulation (FDR = 9.49 × 10^−39^) and processes (FDR = 9.51 × 10^−54^). This cluster included interleukin receptors and the CD antigens *Ccl6*, *Cd44*, *Il20rb*, and *Ciita*. There is little information on the interaction between skeletal muscle and immune systems during fetal development, so the inclusion of immune system-related genes in our analysis of Pax3^+^ cells was a bit surprising. All cells were sorted to a final purity of 97–99% (data not shown), so genes detected in our analysis were unlikely to have originated in non-green cells. As confirmation, we compared our DE genes with those from a similar study by Biressi *et al*.^10^, and found a similar list of immune-related genes such as *Anxa1*, *Cd44*, and *Myb* among others. Previous studies have shown that macrophage infiltration and inflammation occur during satellite cell-mediated skeletal muscle regeneration^33,34^ in adults. Although not a developmental process, many developmental mechanisms are reactivated during adult regeneration of skeletal muscle. It should be noted that these genes were specifically expressed at the latest stage of development that we sampled, E14, after the onset of fetal development, and unlikey to mark angiogenesis.

### Pax3 expression in non-myogenic embryonic forelimb cells

**Figure 3:**
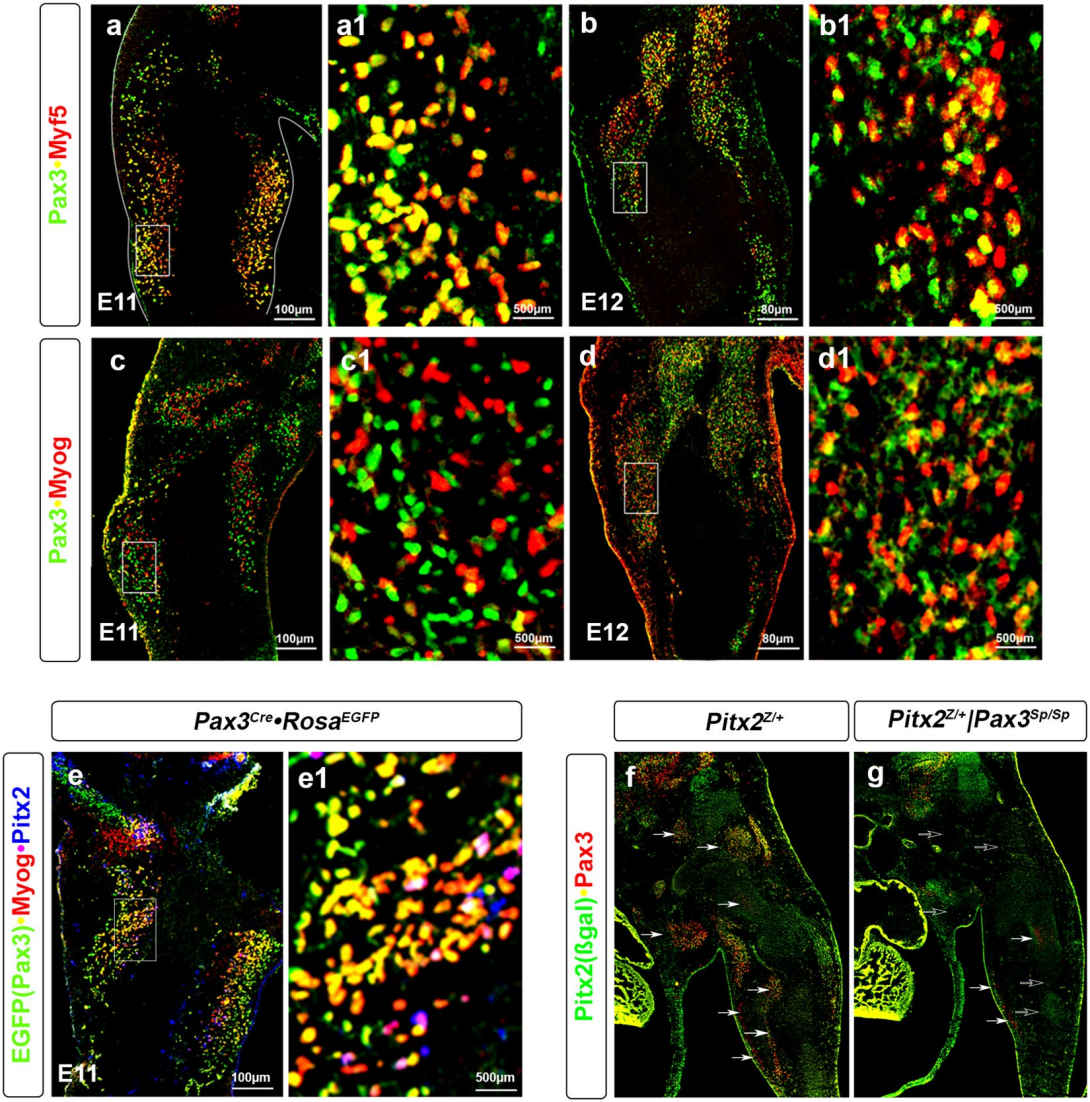
Myogenic and non-myogenic cells populate the *Pax3*^*EGFP*^ lineage in the forelimb. **(a–d)** Immunohistochemistry of E11 **(a**,**a1**,**c**,**c1)** and E12 **(b**,**b1**,**d**,**d1**) forelimb frontal sections of wild type mice for Pax3, Myf5 and Myog. **(a1–d1)** higher magnification of the **a–d** images. **(e)** Immunohistochemistry of E11 forelimb frontal sections of *Pax3*^*EGFP*^ mice for EGFP (Pax3), Myog and Pitx2. **(e1)** higher magnification of the **e** image. **(f**,**g)** Immunohistochemistry of E12 forelimb frontal sections of *Pitx2*^*Z/*+^ **(f)** and *Pitx2*^*Z/*+^*|Pax3*^*Sp/Sp*^ **(g)** mouse for ß-gal(Pitx2) and Pax3.

To further investigate the gene expression profiling findings, we performed double labelled immunohistochemistry for Pax3 and the myogenic markers Myf5 and Myog (Figs. 3a–d1) in forelimbs from E11 and E12 mice. Myf5 marks skeletal muscle cells and brown and white adipocytes^35^, while Myog marks cells committed to the myogenic lineage^36^. Pax3^+^ cells largely overlap with the Myf5^+^ in skeletal muscle at E11 (Fig. 3a,a1) and both brown fat and subcutaneous white fat^37^. At E12, three distinct cell populations were detected: Pax3^+^, Myf5^+^ and Pax3^+^Myf5^+^ double positive (Fig. 3b,b1). At E11, only a small Pax3^+^Myog^+^ cell population was detected (Fig. 3c,c1). By E12, cells are committed to the muscle cell lineage and the Pax3^+^Myog^+^ cell population was enlarged (Fig. 3d,d1). Similarly, triple labeling immunohistochemistry on E11 *Pax3*^*EGFP*^ forelimbs using antibodies against EGFP (to mark the Pax3-derived cells), Myog and Pitx2 (to mark the skeletal muscle cells) was performed^38^ (Fig. 3e,e1). Several cell populations were detected besides the Pax3^+^Myog^+^ and Pax3^+^Pitx2^+^ populations, suggesting the presence of non-myogenic cell types within the *Pax3*^*EGFP*^ lineage. Immunohistochemistry on E12 *Splotch* (*Pax3*^*Sp*^) mice, a natural mutation of the Pax3 locus that results in ablation of skeletal muscle in the forelimbs, also indicated the presence of a small population of Pax3^+^Pitx2^−^ cells in the forelimb (Fig. 3f,g), further supporting the observations that Pax3-derived cells populate other lineages. These observations were in accord with previous studies showing that a subset of *Pax3*^*EGFP*^ cells in the forelimb differentiate into vascular epithelial cells^39^. In limb formation, the vascular and nervous systems develop in parallel to the skeletal system^40,41^, and the Pax3 lineage is likely to give rise to cells that will populate different systems^42,43^.

### Construction of co-expression network during forelimb development

To observe the biological network underlying forelimb development we performed a co-expression analysis, using differentially expressed (DE) genes. A single co-expression network was constructed from pairwise correlation coefficients between each of 4,481 DE genes, using all samples. We opted to construct a single network for all biopsies, rather than state-specific individual networks, to increase the power of our analysis. We focused only on genes that were DE between consecutive developmental states to highlight the genes of most biological relevance, and to decrease computational time. Upon calculating Pearson correlation coefficients (PCC) in a pairwise manner, we determined a FDR cutoff for significant correlation using the following rationale. The *p*-value choice reflected the condition that the node-degree distribution of biological networks closely follows a scale-free distribution^14^. We plotted the *p*-value cutoff vs. the *R*^2^ of a best-fit power line for the resulting node-degree distribution and observed that our co-expression network fits a scale-free topology well (Fig. S2a), allowing the choice of a *p*-value cutoff of 1E-16 resulting in an *R*^2^ cutoff of 0.88. The resulting network had a scale-free degree distribution (Fig. S2b) and a giant component comprising the vast majority (97%) of the nodes, consistent with previous studies of gene regulatory networks^44^. Ultimately, a network with 682 nodes and 3,655 edges was generated, with an average node degree of 10.7.

**Figure 4:**
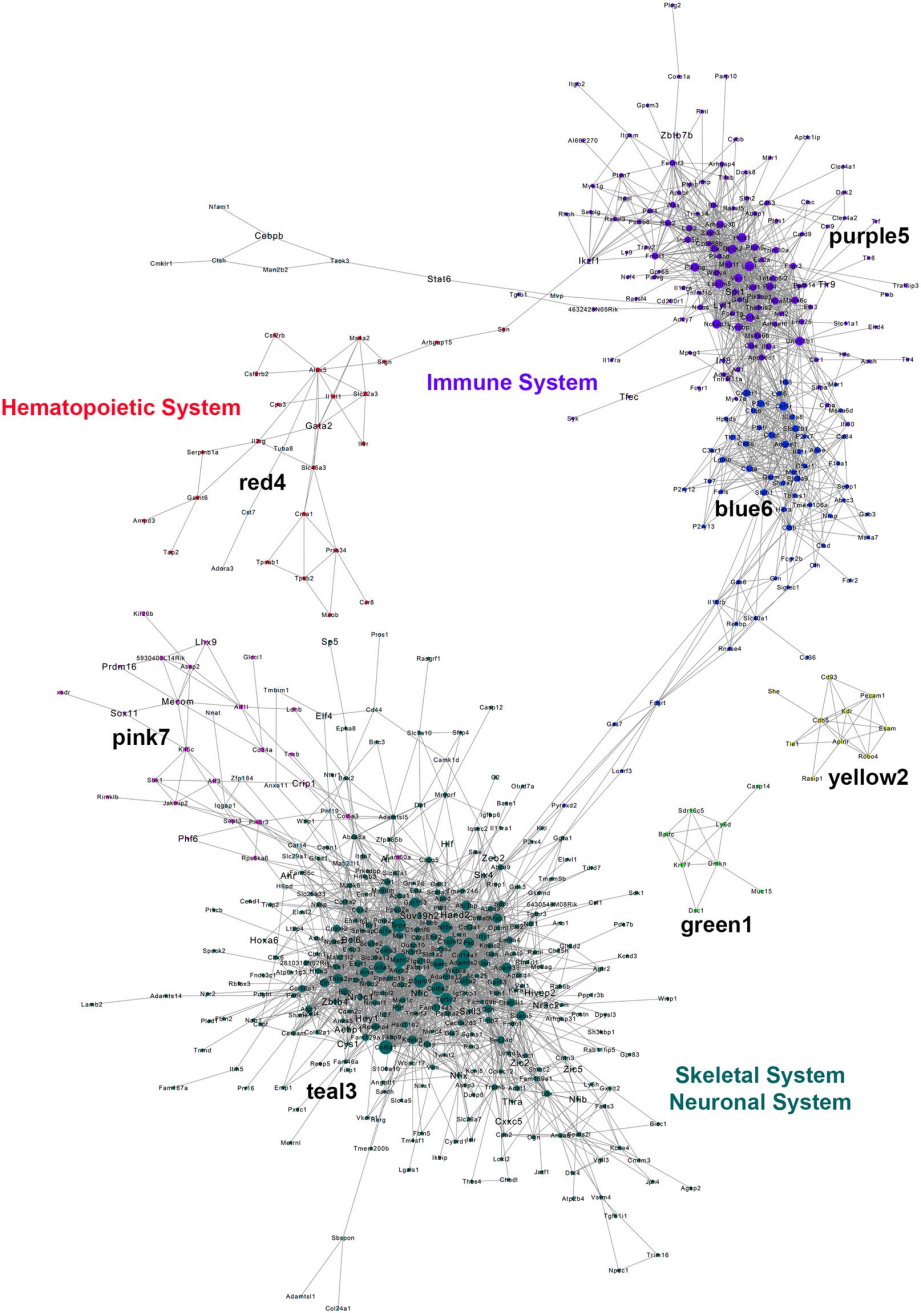
Co-expression network and module identification of the *Pax3*^*EGFP*^ lineage in the forelimb. The generated co-expression network was visualized in Cytoscape software. Nodes (transcripts) are shown as circles, with size proportional to the degree of the node (i.e., the number of neighbor nodes to which it is connected in the network). Seven modules with at least nine nodes were identified via markov clustering, and are color-coded accordingly. The full co-expression network is comprised of two, mostly-distinct subnetworks.

When the network was graphically visualized with Cytoscape software^45^, we observed a single network composed of two mostly independent subnetworks, with smaller individual networks present (Fig. 4). Each node (circle) represents a gene transcript, and edges represent significant correlation between the transcripts. GO term enrichment analysis revealed an overrepresentation of cytoskeletal, skeletal and neuronal system-related genes (skeletal system, neuronal system), and immune response-related genes (immune system, hematopoietic system). The strong presence of the immune and skeletal system-related genes implied that two different transcriptional co-expression networks co-exist during forelimb development with little interaction between them. To identify modules, which are clusters of highly interconnected nodes that together perform a specific biological function^16^, we used the MCL package in R statistical software and performed Markov clustering^46^. Markov clustering identifies modules by simulating flow in networks, and determining the clusters in which the most flow accumulates. However, the weakness of this method was that it assigned each gene to only a single module, which rarely reflects the true underlying biology. Using a module size cutoff of eight, markov clustering identified seven modules, marked by number and color. Two modules (blue6, purple5) comprise one subnetwork, and two (pink7, teal3) comprised another subnetwork. The three smaller modules (red4, green1, yellow2) were mostly independent of either subnetwork (Fig. 4).

GO term enrichment identified significant overrepresentation of collagen fibril organization (FDR = 6.38 × 10^−11^), extracellular matrix organization (7.56 × 10^−11^), and skeletal system morphogenesis (FDR = 8.69 × 10^−04^) related genes in the teal3 module, but no significant enrichment in the pink7 module. The teal3 module represented the muscular and skeletal systems during development, and identification of such a module was expected. Most components of the skeletal system were lumped into one module rather than multiple separate modules with more specific functions, suggesting that the skeletal and muscular systems are intertwined and co-dependent during development, and/or share common mechanisms. Immune and defense-related GO terms such as immune system processes (FDR = 3.61 × 10^−5^) and defense response (FDR = 9.52 × 10^−6^) were overrepresented in the blue6 module, while T-cell related GO terms such as mast cell activation (FDR = 2.29 × 10^−9^) and T-cell proliferation (FDR = 5.26 × 10^−8^) were overrepresented in the purple5 module. Unlike the teal3 module, both immune-related modules were expressed most strongly at E14, implying that immune-related genes are expressed most highly later in fetal myogenesis, rather than at the onset.

Of the smaller modules, the yellow2 module showed enrichment in genes related to vasculogenesis (FDR = 1.38 × 10^−2^) and angiogenesis (3.49 × 10^−4^). Unlike the immune system-related modules, the yellow2 module was not connected to the main network (Fig. 4). Since it was such a small module, angiogenesis and vascularization are likely a relatively small part of the *Pax3*^*EGFP*^ lineage in the mouse forelimb. It should also be noted that the network was constructed to represent all states of development. If co-expression of myogenesis and angiogenesis-related genes is strongest during late embryonic or early fetal myogenesis, it would not be detected in the current network analysis. The red4 module showed no significant enrichment in specific GO terms, making its function difficult to assess. Observing the individual genes in the red4 network, the presence of the SSTF *Gata2* points to a possible function related to the hematopoietic system, since *Gata2* has been shown to be a marker of hematopoietic cells in early development^47^. This argument was strengthened by the proximity and weak connection of the red4 module to the purple5 module, rather than the teal3 module, because of the interdependence and co-regulation between the hematopoietic and immune systems^48^. The only module that showed high overall expression during the early developmental states is the pink7 module, which contained the embryonic myoblast-marker genes *Crip1*, *Lhx9*, *Mecom*, *Phf6*, *Prdm16*, and *Sox11*. Also, it needs to be noted that these are RNA-based measurements, and that protein abundances may not correlate exactly with RNA abundances. This is a topic of great interest that could lead to the discovery of unknown and/or novel cell states.

An alternate explanation for the presence of immune-related genes in Pax3^+^ cells during fetal development could be that the GO term enrichment analysis is inherently biased to some degree because it only takes into account the known and annotated functions of genes. Because of the prevalence of pleiotropy in humans as well as the rich hierarchy of functional annotations in the gene ontology, most genes have multiple, if not dozens of annotated biological functions that can be context-dependent based on tissue type or other variables. Genes that have only been studied in only one system are likely annotated with incomplete information in regard to functions in our contexts. Additionally, certain GO terms such as “immune response” are semantically broad and thus somewhat loosely defined. Taken together, a GO term enrichment analysis could include the wrong context of one or more genes, and bias the results in a way that does not reflect the true underlying biology. More stringent biological validation such as immunohistochemistry with known lineage markers, or transgenic mouse KO studies is required to truly determine whether immune-related genes are expressed during fetal myogenesis in Pax3^+^ cells.

Among the questions raised by this analysis, taking into account the limitations described above, were whether the enrichment in immune-related genes during fetal embryogenesis was caused by non-myogenic *Pax3*^*EGFP*^ subpopulations, or whether the skeletal muscle cells were expressing these genes. The former possibility seems plausible, insofar as it is already known that the *Pax3*^*EGFP*^ lineage gives rise to a small population of vascular endothelial cells. Since samples were sorted to 97–99% purity, gene expression was unlikely to be caused by impurities. Co-expression network analysis revealed that the immune-related subnetwork genes were expressed at E11 (Fig. 4), but overall expression did not peak until E14 (Fig. S2C). One possible explanation is that an immune-related subpopulation of the *Pax3*^*EGFP*^ exists at E11, but these cells do not expand in number until significantly until later in development. Another possibility was that there were two separate networks expressed in the same cell type. These questions reveal the power of co-expression analysis, which can detect gene expression patterns even at low levels of expression^49^.

### Gene expression profiling of SSTFs *in the Pax3-*derived cells in developing forelimbs

To identify the genes involved in early forelimb development we cross referenced the 4,481 DE genes with a list of known SSTFs, and identified SSTFs as stage-specific depending on the averaged SDR values. SDR values for all SSTFs in all samples were calculated as mentioned previously, and SDR values were averaged by time point. SSTFs were classified as specific to each developmental state if the average SDR value for a stage was at least 0.4, and was at least 0.3 greater (additively) than the average for any other state. Similarly, SSTFs were classified as specific to two embryonic states if the SDR for each state was at least 0.3, the average of both SDR values was at least 0.4, and the average of both SDR values was at least 0.3 greater (additively) than the average for any other state. Additionally, the mouse genome informatics (MGI) batch tool was used to determine the known expression of stage-specific SSTFs (Table 1).

**Table 1:** Gene expression profiling of SSTF in the forelimb Pax3 lineage during development.

SSTF	Domain/Family	Developmental State	System
**E11**			
Alx4	HB	E10^70^, E14^71^	SS^70^, SS^71^, CNS^71^, IS^71^
Arid3b	ARID, REKLES	E13, P0	CNS^72^
Dlx1	HTH, HB	E13^72^, E14^72^, P0^72^, E14^71^	CNS^72^, SM^71^, VS^71^, HLS^71^, IS^71^
Dlx2	HTH, HB	E13^72^, E14^72^, E14^71^, P0^72^	VS^71^, IS^71^, CNS^72^
Dmbx1	HB	E13, P0^72^, E14^71^	CNS, SS^71^
Dmrta2	DM	E14^71^	CNS, HLS, IS^71^
Emx1	HB	E13, P0	CNS^72^
Etv4	ETS, WHTH	E15, adult	SS^73^, SM^73^
Etv5	ETS, WHTH	E13^72^, E15^73^, P0^72^, Adult^73^,	CNS^72^, SM^73^, SS^73^
Evx1	HB	E10, E12^74^, E13, P0^72^	CNS
Fli1	ETS, WHTH	E10^75^, E11^75^, E14^76^	VS^75^, SM^76^, SS^76^
Gbx2	HB	E14^71^, P0^72^	SS^71^, CNS^72^
Grhl2	CP2	E13, E14	CNS^71^, HLS^71^, IS^71^
Gsc	HB	E14	SM, VS, SS, CNS^71^
**Hand2**	bHLH	E18	A^77^
**Hey1**	OJ, bHLH	E13^72^, E14^78^, E15^79^, E17^78^, P0^72^	CNS^72^
**Hoxa6**	HB	E13^80^	SS^80^, CNS^80^
Hoxa9	HB	E13^72,87^ E14^71^	SS^81^, CNS^72^, VS^71^, SS^71^, IS^71^, CNS^71^
Hoxa11	HB	E13	CNS^72^
Hoxd4	HB	E12^80^, E12^82^, E13^72^, E14^71^, P0^72^	SS^80^, CNS
Hoxd10	HB	E14	CNS^71^
Insm1	ZNF	E14	CNS^71^
Isl2	ZNF, HB	E13, P0	CNS^72^
Lhx1	HB, ZNF	E12^83^, E13^72^, E14^71^, P0^72^	CNS
Mybl	HTH	E13	CNS^72^
Mycn	bHLH	E9, E13^84^, E16^85^	CNS
Nkx3-1	HB	E13	CNS^72^
Nr0b1	NHR	E13^72^, E14^71^, P0^72^	CNS, VS, SS^71^
Pax9	PRD, HB, WHTH	E10^86,87^, E11^86^, E12^86,87^, E13^72^, E14^86–88^, E15^87^, E16^87^, E18^87^, adult^87^	SS, CNS^72,87^
Phf6	ZNF		CNS^87^
**Phf21b**	ZNF	E13, P0	CNS^72^
Pou2f1	POU, HB, LBD	E13	CNS^72^
Pou4f1	POU, HB, LBD	E9^89^, E13, P0^72^	CNS
Ppargc1a	RRM	E13^90^, E17^91^, E18^91^, P0^91^	CNS^90^, A^91^
Rax	HB	E13^72^, E14^71,92^, P0^72^	CNS, SS^71,92^
Sall1	ZNF	E14	CNS^88^
**Sall3**	ZNF	E10^93^, E16, P6^85^	CNS
Sall4	ZNF	E10	CNS^93^
Shox2	HTH, HB	E13^72^, P0^72^, adult^94^	CNS^72^, SM^94^
**Six4**	HB	E13	CNS^72^
**Suv39h2**	CHR	E14	VS, CNS, HLS, IS^71^
Tfap2c	ZNF	E13	CNS^72^
Trps1	ZNF	E11, E12^95^, E13^96^, E14^88,95^	SM^88^, SS, IS
Uncx	HB	E13^97^, E14^71^, adult	SM, SS, VS, CNS, IS^71^
**Zbtb16**	SKP1/BTB/POZ, ZNF	E14	SS, CNS, HLS, IS^71^
**Zic3**	ZNF	E9^98^, E13^72^, E16^85^, P6^85^	SS^85^, CNS
**Zic5**	ZNF	E13, P0	CNS^72^
**E11**			**E12**
Dmrta1	DM	E13	CNS^72^
Evx2	HB	E13, P0	CNS^72^
Hmga1	AT	adult	Adipose^99^
**Hmga2**	AT	E14	SS^88^
Hoxa2	HB	E14	VS, SS, CNS, HLS, IS^71^
**Hoxa3**	HB	E12^80,82^, E13, P0^72^	CNS, SS^80,100^
Hoxa4	HB	E12^80,82^, E13^82^	CNS, SS^82^
**Hoxa5**	HB	E9^101^, E12^80,102^, E13^101^	CNS, SS^80,103^, SM^101^
Hoxa7	HB	E10^93^, E12^100^, E13, P0^72^	CNS, SS^100^
Id1	bHLH	E10-E11^104^, E11^105^, E13^72,104^, E14^105^, E16^85,105^, E18^106^, P0^106^, adult^106^	CNS, VS^105^, SM^106^, SS^104^
**Lhx9**	HB, ZNF	E11-E12^107^, E13^72^, E14^23,71^	CNS, VS^71,92^, HLS^71,92^, IS^71,92^
Nr2f1	ZNF, COUP	E11^108^, E13, P0^72^	CNS
Pitx2	HB	E12-E13^38^, E14^71,88,109,110^, P0^72^adult^38^	CNS, IS^38^, SM SS^71^,VS SM
Pknox2	HB	E10^111^, E12^111^, E14^88^, adult^111^	SM, SS^111^
Six1	HB	E12^112^, E17^113^	SM^112^, VS^113^
Six2	HB	E11^114^ E14^71^, P0^72^	CNS^72^, HLS^71^, SS^114^
**Sox11**	HMG	E8^115^, E9^115^, E14^71^	SS, CNS, HLS, IS^71^, VS^115^
Zfp423	ZNF	E14	VS, CNS, IS^71^
**E12**			
Ebf2	IPT, COE	E11^116^, E13, P0^72^	CNS
Esrrg	ZNF	E10^117^, E13^72,117^, E18^118^, P0^72^, adult^119^	CNS, SM^119^
Fezf2	ZNF	E13, P0	CNS^72^
Foxa1	FH, WHTH	E10^120^, E13^72^, E14^71^, P0^72^	CNS
Hoxc5	HB	E12^80,121^, E13^72^	CNS, SS^80^
Meox1	HB	E12, E14	SM^122^
Onecut2	HB, CUT, LBD	E9	CNS^123^
Pitx1	HB	E14^71^	SM, SS, CNS, IS, VS
Wt1	ZNF	E13^124^, E14^71^	SM^124^, VS, SS, CNS, HLS, IS^71^
**E13**			
Egr4	ZNF	E13, P0	CNS^72^
**E13**			**E14**
**Aebp1**	AEBP1/CPX	E10, E11, E13^125^, E14^71^, E15^126^, E16, adult^125^	A, CNS^71^, SS, VS
**Ahr**	bHLH	E13, E15^127^	CNS^72^ SM^127^
**Ar**	ZNF	E12	IS^128^
Arid3b	ARID, REKLES	E13, P0	CNS^72^
**Bcl6**	SKP1/BTB/POZ, ZNF	P0^72^, adult^129^	CNS^72^, SM^129^
Foxf2	FH, WHTH	E10^130^, E11^130^, E12^130^, E14^88^	SS^88^, VS^130^
Foxq1	FH, WHTH	E13, P0	CNS^72^
Foxs1	FH, WHTH	E11	VS^131^
**Hivep2**	ZNF	E13	CNS^72^
**Hlf**	bZIP	E14	VS, SS, CNS, IS^71^
Hoxd8	HB	E12^132^, E13^72^, adult^133^	CNS
Jdp2	bZIP	E14	SS^88^, SM^133^
Klf14	ZNF	adult	SM, A^134^
Mitf	bHLH, MiT/TFE	adult	SM^135^
**Nfic**	CTF/NFI, MAD	E12^136^, E13^72^, E14^136^, P0^72^, adult^136^	CNS^72^, SM^136^
**Nfix**	CTF/NFI, MAD	E12^136^, E13^72^, E14^71,136^, P0^72^, adult^136^	CNS, VS^71^, SS^71^, HLS^71^, IS^71^, SM^136^
**Nr3c1**	ZNF	E13^72^, E14^137^, E15^137^, E16^137^, P0	CNS^72^, SM^137^
**Nr3c2**	NHR	E13, P0	CNS^72^
Nr4a1	ZNF	P0	CNS^72^
Plagl1	ZNF	E9^138^, E10^138^, E11^139^, E13^139^, adult^140^	CNS^138^, SM, VS^139^
Ppara	ZNF	E17-E18, P0	A^91^
**Runx3**	AML1, p53/RUNT	E13, E14^71^, P0	CNS^72^, SS, HLS, IS^71^
**Thra**	ZNF	E13^72^, E14^71^, E17^91^, E18^91^, P0^72,91^	A^91^, CNS, VS^71^, SS^71^, HLS^71^, IS^71^
Thrb	ZNF	E17, E18, P0	A^91^
Vdr	ZNF	E14	SS, CNS, IS^71^
**Zbtb4**	SKP1/BTB/POZ, ZNF	adult	VS, A^134^
**Zeb2**	HB, ZNF	E14	VS, CNS, HLS, IS^71^
**E14**			
Ascl2	bHLH	E14	VS, SS, CNS, IS^71^
Cebpa	bZIP, C/EBP	E16^91,141^, E17^91^, E18, P0^91,141^	A
**Cebpb**	bZIP, C/EBP	E14^71^, E16^91^-E18^91^, P0^91^, adult^91^	A^91^^,^ IS^71^
Cebpd	bZIP, C/EBP	E16-E18, P0	A^91^
Eaf2	EAF	adult	SM^142^
Elf1	ETS, WHTH	E13^72^, E14^71^, P0^72^	CNS, SS^71^, HLS^71^, IS^71^
**Elf4**	ETS, WHTH	E14	VS, SS, CNS, HLS, IS^71^
Gata1	ZNF	E8^143^, E14^71^	B^143^^,^ VS, SS, CNS, HLS, IS^71^
**Gata2**	ZNF	E10-E11^144^, E13^72^, E14^71^, P0^72^	CNS, VS, HLS, IS^71^
Hoxc9	HB	E11, E13	SS^145^
**Ikzf1**	ZNF	E13^72^, E14^71^, P0^72^	CNS, HLS^71^
Klf2	ZNF	E12^146^, E14^71^, adult^147^	VS, SM^147^, SS^71^
Klhl6	SKP1/BTB/POZ	E8	VS^148^
Pou2f2	POU, HB	E10, E12^149^, E13^72^	CNS
Pparg	ZNF	E14^71^, E16^141^, E17^91^, E18^91,141,150^, P0^91^, adult^134,151^	SA, S^71^, CNS^71^
**Spi1**	ETS, WHTH	E14	SS, CNS, HLS, IS^71^
Stat5a	SH2, STAT	E13, E14^71^	CNS^72^, HLS^71^
Tal1	bHLH	E7 - E11^152,153^, E13^72^, E14^71^, E15^153^	B^152,53^, CNS^72^, SS^71^, VS
**Tfec**	bHLH	E13	CNS^72^
**Zbtb7b**	SKP1/BTB/POZ, ZNF	E13, P0	CNS^72^

A: adipose; B; blood; CNS: central nervous system; SM: skeletal muscle; SS: skeletal system; HLS: hemolymphoid system; ISS: integumentary system; VS: vascular system; ARID: AT-rich interaction domain; AT: AT-hook; bHLH: basic helix-loop-helix; BTB: BR-c, ttk, and bab domain; bZIP: basic leucine-zipper; C/EBP: CCAAT/enhancer-binding protein domain; CHR: chromo domain; CP2: connective peptide 2 domain; CPX: carboxypeptidase domain; CUT: CUT domain; ETS: erythroblast transformation specific domain; FH: forkhead box; HB: homeobox; HMG: high-motility group; HTH: helix-turn-helix; LBD: lambda domain; NHR: nuclear hormone receptor; OJ: orange domain; POZ: pox virus and zinc finger domain; PRD: proline-rich domain; RRM: RNA-recognition motif; SH2: Src homology 2 domain; STAT: signal transducer and activator of transcription domain; WHTH: winged helix-turn-helix; ZNF: zinc finger. SSTFs indicated as bold are found in networks.

SSTFs expressed specifically at E11 belonged to homeodomain (HD) and/or zinc-finger (ZNF) transcription factor families, were primarily expressed in the CNS during early-mid gestation (Table 1) and are nodes of the network (Fig. 4) with few expressed specifically in skeletal muscle. This further supports the previous observations that multiple Pax3^+^ non-myogenic cells exist within the *Pax3*^*EG*FP^ lineage. SSTFs expressed at both E11 and E12 follow the same trends, except were composed mostly of homeodomain SSTFs. No SSTFs expressed specifically at E12 or E13 were present in the co-expression network, implying that they may perform stage-specific roles. Expression of immune-related genes occurs later in fetal embryogenesis. SSTFs expressed specifically at E13 and E14 belonged to the ZNF family, with only *Zeb2* and *Pou2f2* possessing a homeodomain. While most of the SSTFs expressed at E13 and E14 are expressed in the CNS, they are also expressed in other tissue types such as the vascular system (VS) and hemolymphoid systems (HLS), among others, and were present in the co-expression network in skeletal system, nervous system, and immune system subnetworks. SSTFs with established immunogenic functions included *Bcl6*, *Ikzf1*, and *Zbtb7b* among others. *Bcl6* is also part of the skeletal and nervous system subnetwork, meaning it may not have an immune-specific role in this context.

### Gene expression profiling of signaling molecules *in the Pax3-*derived cells in developing forelimbs

During development, signaling molecules convey information to cells about their direction, behavior and specification by activating transcriptional programs. Cell shapes and cytoskeletal changes regulate cell lineages and organ formation. *Notch*, *Hedgehog (Hh*), *Wingless/Wnt*, *Bmp* (bone morphogenetic proteins), *Egf* (epidermal growth factor), and *Fgf* (fibroblast growth factor) signaling can generate morphogen gradients across varying distances that pattern cells in a concentration-dependent manner.

**Figure 5:**
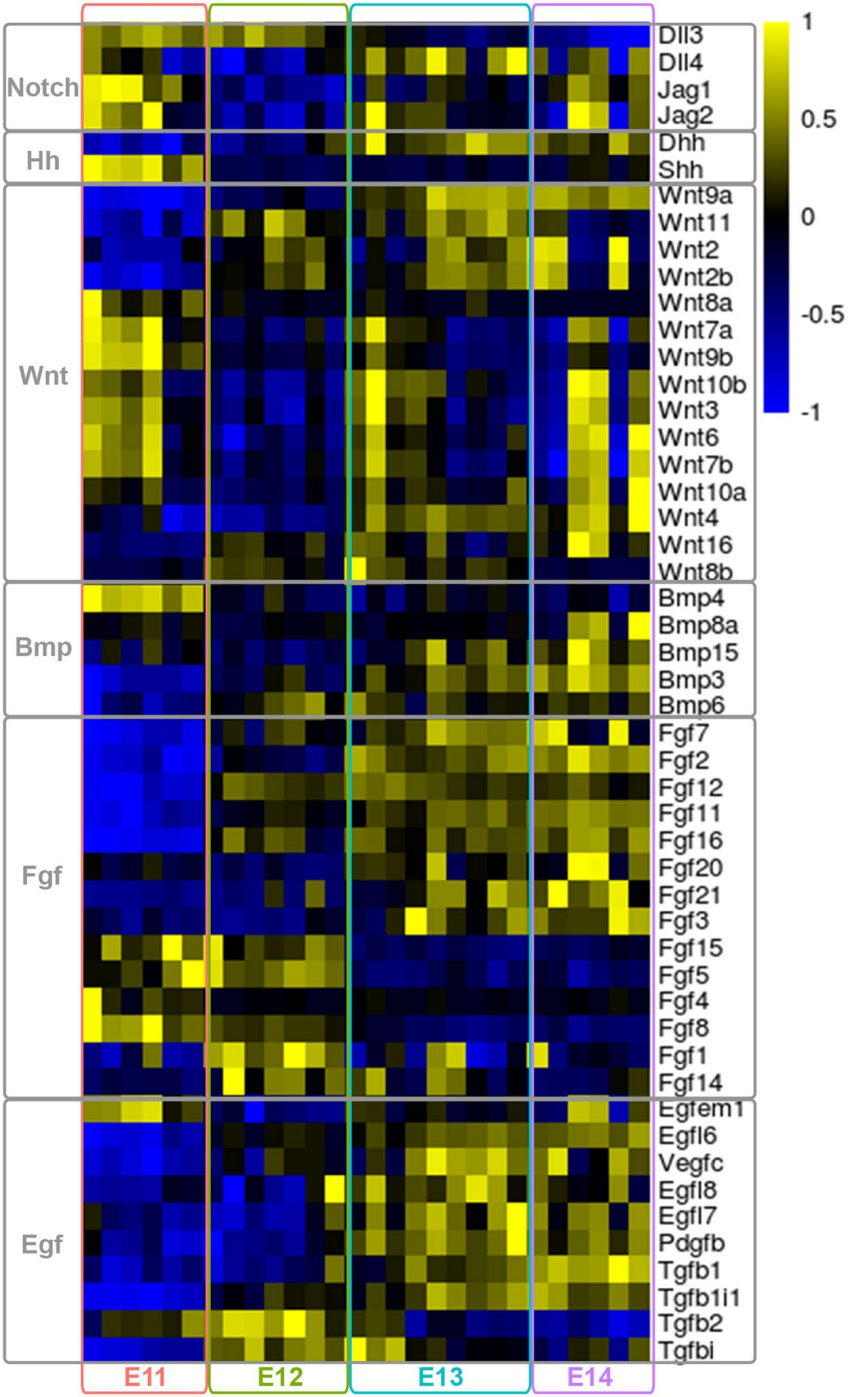
Relative expression of known signaling ligands. Relative expression of known signaling ligands relevant in forelimb development was visualized as a heatmap using SDR values, with columns representing samples ordered by stage, and rows representing ligands. Yellow indicated high gene expression relative to the average of all samples, and blue indicates low expression.

Notch functions in organ formation during development, including somitogenesis, as well as in adult homeostasis by determining cell fate and maintaining pluripotency^50^. Members of the Notch pathway were highly expressed in the *Pax3*^*EGFP*^ cells in the embryonic forelimb (E11, Fig. 5). The elevated *Shh* levels in the *Pax3*^*EGFP*^ lineage at E11 were in accord with its expression at the posterior margin of limb buds between E9–E12^51^. Shh is produced by cells located in the zone of polarizing activity (ZPA) in the mesenchyme and regulates patterning along the anterior-posterior axis. Shh signaling is also involved in timing myogenic differentiation, promoting slow muscle differentiation, and controlling migration into the distal part of the limb^52,53^. Wnts are secreted proteins that control a multitude of diverse developmental processes. At the onset of limb development, the limb buds form as a result of an interplay between Fgf  and Wnt signaling (E11, Fig. 5). Wnt proteins control the morphogenesis of specific tissues in the limb such as musculature, synovial joints, cartilage, and bone. Wnt6 from the limb ectoderm promotes limb myogenesis via Pax3 and Myf5^54^. Wnt7a maintains the expression of N-cadherin, which is essential for myogenic migration and chondrogenesis^55^. Wnt3a induces the apical ectodermal ridge (AER) formation and Fgf8 expression through the canonical Wnt pathway^56^. Wnt4 and Wnt11 are expressed in the mesenchyme surrounding the developing cartilage elements which form at the onset of fetal development (E13, E14, Fig. 5). Fgfs , produced by the AER (Fgf2, Fgf4, Fgf8, Fgf9) and in the underlying mesenchyme (Fgf2, Fgf10), are required for proximal-distal outgrowth^57^. Specification of the vascular and hematopoietic systems is a characteristic of fetal development, following the patterning of the skeletal system characterized by expression of members of the Egf  signaling pathway (E12–E14, Fig. 5). These data collectively suggest that interactions of muscle, bone, cartilage, tendon, and ligament are critical for the correct assembly of the musculoskeletal system during development, which is further enhanced by the development of the immune and hematopoietic systems.

## Conclusion

FACS-Seq analysis of embryonic and fetal *Pax3*^*EGFP*^ cells identified many transcripts outside the myogenic lineage. *Pax3*-derived cells populated the musculoskeletal, vascular, neuronal, immune and hematopoietic lineages. These findings suggest that the dermomyotome *Pax3*-derived cells may have the characteristics of a stem cell niche that can give rise to several lineages to form a functional forelimb, and provides a framework for future single cell sequencing within the forelimb during development.

## Materials and Methods

All methods were carried out in accordance with relevant guidelines and regulations. All experimental protocols were approved by the Environmental Health and Safety Committee at Oregon State University. All animal experiments were performed in accordance to institutional and National Health and Medical Research Council guidelines. The experimental protocol was approved by the Institutional Animal Care and Use Committee at Oregon State University.

### Fluorescence assisted cell sorting (FACS) of embryonic mouse forelimb cells

Mice were fed the standard PicoLab Rodent Diet 20, 5053*, a managed formulation delivers constant nutrition. Female ICR mice were plugged on consecutive days by male *Pax3*^*Cre*^*|Rosa*^*EGFP*^ mice. At 11, 12, 13, and 14 days post vaginal plug, female mice were euthanized, and embryos collected in PBS over ice and embryos were genotyped under a fluorescent microscope. Forelimbs were dissected between the caudal edge of the shoulder and the lumbar region. Isolated forelimbs from each litter were pooled in Dulbecco’s Modified Eagle Medium (DMEM) with 4.5 g/L glucose, based on *Pax3*^*Cre*^*|Rosa*^*EGFP*^ positive (green, G) and negative (white, W) genotypes. Dissociation of embryonic forelimbs was carried out as described previously^58^ with the following modifications. DMEM was removed, and dissociation buffer (HBSS without CaCl_2_, MgCl_2,_ MgSO_4_ (Gibco), 2 mg/mL Type I Collagenase (Worthington Biochem), 5 mM EDTA was added, ~6 forelimbs per 1 mL buffer for E11 and E12, and ~2 forelimbs per 1 mL buffer at E13 and E14. Forelimbs were incubated for 3 minutes at 37 °C, and pipetted 10 times through a 1 mL pipette tip to promote dissociation. Forelimbs were incubated and pippeted once more at E11, E12, and E13, and twice more at E14. After the final dissociation step, each pooled sample was centrifuged at 5,000 rpm for one minute. The media was aspirated, cells were resuspended in PBS by pipetting 15 times, to a final concentration between 1 × 10^6^ and 1 × 10^7^ cells/mL. Cell suspensions were passed through a 35 µm nitex filter again before they were sorted. Cell suspensions were sorted using a Sony SH800 cell sorter (Sony Inc). EGFP^+^ (G) cells were sorted directly into PBS. Once the full samples have been sorted, each tube (G) was spun at 3800 rpm for 15 minutes at 4 °C. PBS was aspirated off the cell pellets, and cell pellets were lysed with 350 µL Buffer RLT with added ß-mercaptothanol (Qiagen). Lysates were kept over ice until all samples were sorted.

### RNA preparation, sequencing and analysis

RNA was extracted using RNAeasy mini kit (Qiagen) following the manufacturer’s protocol. RNA was tested for quality and degradation using the AATI Fragment Analyzer (ATI). RNA libraries were sequenced on a 100 bp single-end run on the Illumina Hiseq. 4000 (Illumina, San Diego, CA). Library preparation was done by trained technicians at the GC3F core facility using the Kapa Biosystems Stranded mRNA-Seq Kit (Kapa). Libraries were created and sequenced, corresponding to six (E11.5), seven (E12.5), nine (E13.5), and six (E14.5) biological replicates. Primary Illumina data image analysis, base calling, and read-quality filtering were done using the Casava pipeline version 1.8.2 (Illumina). Each sample was processed and analyzed with the same methods. After filtering low quality reads TopHat version 2.1.0 was used to align all reads to the mm10 genome with default parameters and to identify splice junctions^59,60^. HTseq was used to create count tables from tophat2 aligned reads^61^. DESeq2 was used to calculate differential gene expression between time points^62^ using an FDR adjusted cutoff of *p* ≤ 0.05, with a fold change ≥1.5, between any two consecutive time points. Principal component analysis was performed using the prcomp function in R software^63^. Heatmaps were generated using the pheatmap package in R software^64^. Signed difference ratios (SDR) were calculated similar to^65^, except the average for each gene across all samples was subtracted from each sample. Fastq sequences were deposited to the NCBI gene expression omnibus (GEO) sequence read archive (SRA) under the accession SRP126903.

### Co-expression Network Construction and Analysis

Co-expression networks were constructed as previously described^18^. Pairwise correlation coefficients were calculated between each of 4,481 identified DEGs, in all samples, sing an adjusted FDR cutoff of p ≤ 1e-16. The co-expression network was visualized in Cytoscape^45^, and modules were identified via markov clustering^66^ using the package MCL in R software. GO term enrichment in modules was determined by Panther GO^67,68^. R software custom code used for co-expression analysis is available in supplemental code file.

### Immunohistochemistry

Immunohistochemistry was performed as previously described^69^.

## Electronic supplementary material

Supplementary information
