# Supplementary material for: FACS-Seq analysis of Pax3-derived cells identifies non-myogenic lineages in the embryonic forelimb
Source: Sci Rep. 2018 May 16;8:7670. doi: 10.1038/s41598-018-25998-1 (PMC5956100; doi:10.1038/s41598-018-25998-1)
Supplement: Supplementary file 1 — Supplementary information [file 41598_2018_25998_MOESM1_ESM.pdf]

Supplementary Information

FACS-Seq analysis of *Pax3*-derived cells identifies non-myogenic lineages in the embryonic forelimb

Arun J. Singh[1], Chih-Ning Chang[1,2], Hsiao-Yen Ma[1], Stephen A. Ramsey[3,4], Theresa M. Filtz[1], Chrissa Kioussi[1,5]

[1]Department of Pharmaceutical Sciences, College of Pharmacy; [2]Molecular Cell Biology Graduate Program; [3]Department of Biomedical Sciences, College of Veterinary Medicine; [4]School of Electrical Engineering and Computer Science, Oregon State University, Corvallis, Oregon, 97331, USA; [5]corresponding author: chrissa.kioussi@oregonstate.edu; TEL 541-7372179

```
#Clear data and load packages
rm(list = ls())
source("https://bioconductor.org/biocLite.R")
library(ggplot2)
library(DESeq2)
library(pamr)
library(MCL)

#Set working directory
setwd("/home/arun/uo_pax3/only_new/newest/embvfet/")
directory <- "/home/arun/uo_pax3/only_new/newest/embvfet/"

#Set up DESeq2 data, based on names of HTSeq counts in working directory
sampleFiles <- dir(pattern = 'sorted')
stageMatch <- regexpr(pattern = 'E1[0-9]', dir(pattern = '.txt'))
sampleStages <- regmatches(dir(pattern = '*.txt'), stageMatch)
sampleTable <- data.frame(sampleName = sampleFiles, fileName = sampleFiles, stage =
sampleStages)

#Calculate DESeq2 from HTSeq count tables
ddsHTSeq <- DESeqDataSetFromHTSeqCount(sampleTable = sampleTable, directory = directory,
design = ~ stage)
#Filter out genes with zero counts
ddsHTSeqFiltered <- ddsHTSeq[ rowSums(counts(ddsHTSeq)) > 1, ]
ddsHTSeqFiltered <- DESeq(ddsHTSeqFiltered)

#Generate log2 normalized count matrix
rld <- rlog(ddsHTSeqFiltered, blind = F)
logTransCounts <- assay(rld)

#Calculate Principle component analysis based on log2 normalized count matrix
OGPCAN <-prcomp(logTransCounts, center = T, scale. = T)
OGPCAN_matrix <- as.data.frame(OGPCAN$rotation)
OGPCAN_matrix <- OGPCAN_matrix[, 1:2]
OGPCAN_matrix$Stage <- c(rep("E11", 6), rep("E12", 4), rep("E13", 9), rep("E14", 6))

#Plot PCA
ggplot(OGPCAN_matrix, aes(PC2, PC1, color = Stage)) +
  geom_point(size = 3) +
  theme(axis.text.x = element_text(size = 14, color = "black"),
        axis.title.x = element_text(size  = 16, face = "bold"),
        axis.text.y = element_text(color = "black", size = 14),
        axis.title.y = element_text(size = 16, face = "bold"),
        legend.title = element_text(size = 16, face = 'bold'),
        legend.text = element_text(size = 14)) +
  scale_color_discrete(name = "Stage")

#Calculate differentially expressed genes from DESeq2
E11.5_E12.5_final <- results(ddsHTSeqFilteredNew, contrast = c("stage", "E11", "E12"))
E12.5_E13.5_final <- results(ddsHTSeqFilteredNew, contrast = c("stage", "E12", "E13"))
E13.5_E14.5_final <- results(ddsHTSeqFilteredNew, contrast = c("stage", "E13", "E14"))
```

```
#Set FDR threshold of p <= 0.05 and fold change >= +-1.5
e11.12.res <- as.data.frame(as.matrix(subset(E11.5_E12.5_final, padj < 0.05)))
e11.12.res$absFC <- abs(e11.12.res$log2FoldChange)
e11.12.res <- subset(e11.12.res, absFC >= log(1.5, 2))
e11.12.res <- e11.12.res[order(e11.12.res$padj), ]

e12.13.res <- as.data.frame(as.matrix(subset(E12.5_E13.5_final, padj < 0.05)))
e12.13.res$absFC <- abs(e12.13.res$log2FoldChange)
e12.13.res <- subset(e12.13.res, absFC >= log(1.5, 2))
e12.13.res <- e12.13.res[order(e12.13.res$padj), ]

e13.14.res <- as.data.frame(as.matrix(subset(E13.5_E14.5_final, padj < 0.05)))
e13.14.res$absFC <- abs(e13.14.res$log2FoldChange)
e13.14.res <- subset(e13.14.res, absFC >= log(1.5, 2))
e13.14.res <- e13.14.res[order(e13.14.res$padj), ]

#List all genes DE between any two sequential stages
all.de.genes <- unique(c(rownames(e11.12.res), rownames(e12.13.res), rownames(e13.14.res)))

#Order samples by approximate order from PCA plot
sample.order.1 <- c(1,2,3,4,5,6,7,8,9,10,12,13,14,15,16,17,18,19,11,20,21,22,23,24,25)
sample.order.2 <- c(5,3,4,6,2,1,7,9,8,10,14,12,15,17,18,16,13,11,19,20,21,25,24,22,23)

logTransCountsOrdered <- logTransCounts[, sample.order.1]
logTransCountsOrdered <- logTransCounts[, sample.order.2]

#Correlation analysis

#Function to remove genes with 0 variance between samples

var_filter <- function(df) {
  select_vec <- logical()
  for(i in 1:length(rownames(df))) {
    if(sd(df[i,]) == 0) {
      select_vec <- append(select_vec, F)
    } else {
      select_vec <- append(select_vec, T)
    }
  }
  return(df[select_vec,])
}

#Create normalized count table of only DE genes of interest
sig.counts <- logTransCountsOrdered[match(all.de.genes, rownames(logTransCountsOrdered)), ]

#Remove genes with 0 variance
sig.counts.filtered <- var_filter(sig.counts)

#Function to calculate pairwise correlation between each gene in a data frame
#Input is a data frame or matrix with genes as rows and samples as columns
#Output is a data frame with four columns
#Gene1, P-value adjusted with fdr, correlation (pearson correlation coefficient), Gene2
correlation_test <- function(df1) {
```

```
  cor_vec <-numeric() #Set vectors to be used to reduce memory use
  p_vec <- numeric()
  whole_vec <- numeric()
  name_vec <- character()
  final_df <- data.frame()
  count_r1 <- 1
  while(count_r1 <= length(rownames(df1)) - 1) {
    for(i in 1:(length(rownames(df1)) - count_r1)) {
      whole_vec <- as.numeric(cor.test(as.matrix(df1[count_r1,]), as.matrix(df1[i+count_r1,]), 't',
'pearson', exact=NULL)[1:5])
      cor_vec <- append(cor_vec, whole_vec[4])
      p_vec <- append(p_vec, whole_vec[3])
      name_vec <- append(name_vec, rownames(df1)[i + count_r1])
    new_pvec <- p.adjust(p_vec, 'fdr')
    og_name_vec <- rownames(df1)[count_r1]
    new_df <- data.frame(name_vec, new_pvec, cor_vec)
    new_df$og_name_vec <- og_name_vec
    final_df <- rbind(final_df, new_df)
    p_vec <- numeric()
    count_r1 <- count_r1 + 1
    name_vec <- character()
    cor_vec <- numeric()
  }
  colnames(final_df) <- c("Gene2", "pAdj", "Correlation", "Gene 1")
  final_df <- final_df[final_df$pAdj <= 0.1,]
  return(final_df)
}

#calculate correlation dataframe
corr_df <- correlation_test(sig.counts.filtered)

#Create a function that fits a power law to the correlation dataframe calculated earlier
#Power fit
powerFit <- function(df, p.vec) {
#Pre-define vectors for more efficient memory
  r.sq.vec <- numeric()
  p.thresh.vec <- numeric()
  edge.vec <- integer()
  node.vec <- integer()
  ave.deg.vec <- numeric()
  for(i in 1:length(p.vec)) {
#Iterate through the p-value vector and calculate network statistics
    new.df <- subset(df, pAdj <= p.vec[i])
    tmp.genes <- c(as.character(new.df$Gene2), as.character(new.df$`Gene 1`))
    n.nodes <- length(unique(tmp.genes))
    n.edges <- length(new.df$Gene2)
    ave.deg <- 2 * n.edges / n.nodes
    nodes <- table(as.integer(table(tmp.genes)))
    node.df <- data.frame(as.integer(names(nodes)), as.integer(nodes))
    colnames(node.df) <- c("degree", "nodes")
    tmp.mod <- lm(formula = log(degree, 10) ~ log(nodes, 10), data = node.df)
    r.squared <- as.numeric(summary(tmp.mod)[8])
    r.sq.vec <- append(r.sq.vec, r.squared)
```

```
    p.thresh.vec <- append(p.thresh.vec, p.vec[i])
    edge.vec <- append(edge.vec, n.edges)
    node.vec <- append(node.vec, n.nodes)
    ave.deg.vec <- append(ave.deg.vec, ave.deg)
    new.df <- data.frame()
  }
  df.final <- data.frame(p.thresh.vec, node.vec, edge.vec, ave.deg.vec, r.sq.vec)
  colnames(df.final) <- c("Pval", "Nodes", "Edges", "AveDegree", "R.squared")
  return(df.final)
}

#Create a function that generates a vector of p values to be used to determine an appropriate cutoff
for generating a coexpression network
pseq <- function(start, long) {
  count = 1
  p.vec <- numeric()
  new.num <- start
  while(count <= long) {
    new.num <- new.num/2
    p.vec <- append(p.vec, new.num)
    new.num <- new.num/5
    p.vec <- append(p.vec, new.num)
    count = count + 1
  }
  return(p.vec)
}

pvec <-pseq(0.1, 30)
#Create a dataframe with R^2 values vs. p-value cutoff threshold
model.fit <- powerFit(cor_df, pvec)


#Create correlatin dataframe based off powerfit to be exported to cytoscape
finalCorrDf <- subset(cor_df, pAdj <= 1e-16)
```

Figure S1

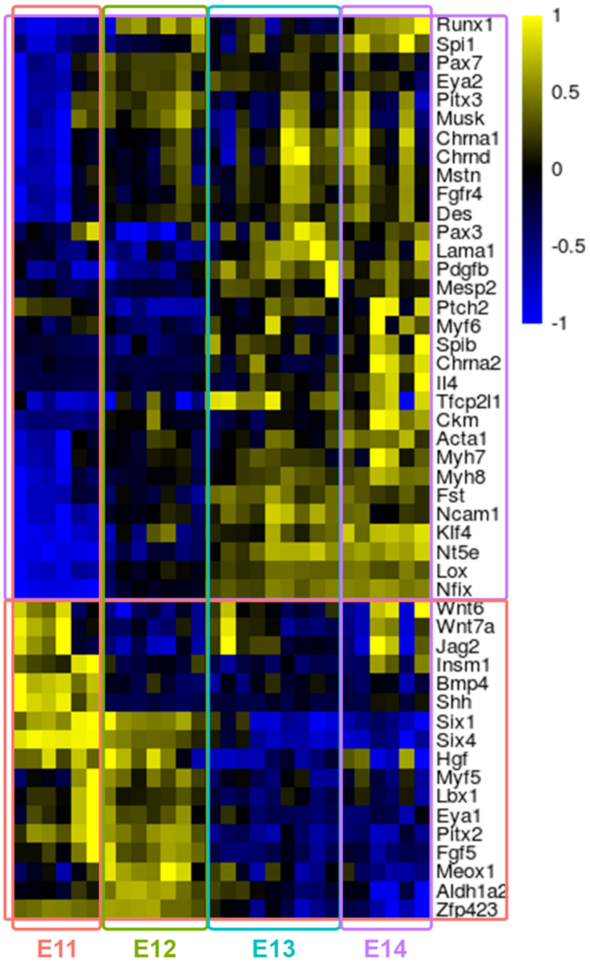

Figure S1: Expression profiling of known genes of the *Pax3*^{EGFP} lineage in the forelimb

Heatmap constructed using SDR values of genes from the literature with known expression patterns. Columns represent biopsies from four developmental states, E11, E12, E13 and E14. Rows represent genes. Genes were cluster based on expression in embryonic or fetal development, and represent genes involved in embryonic and fetal myogenesis.

Figure S2

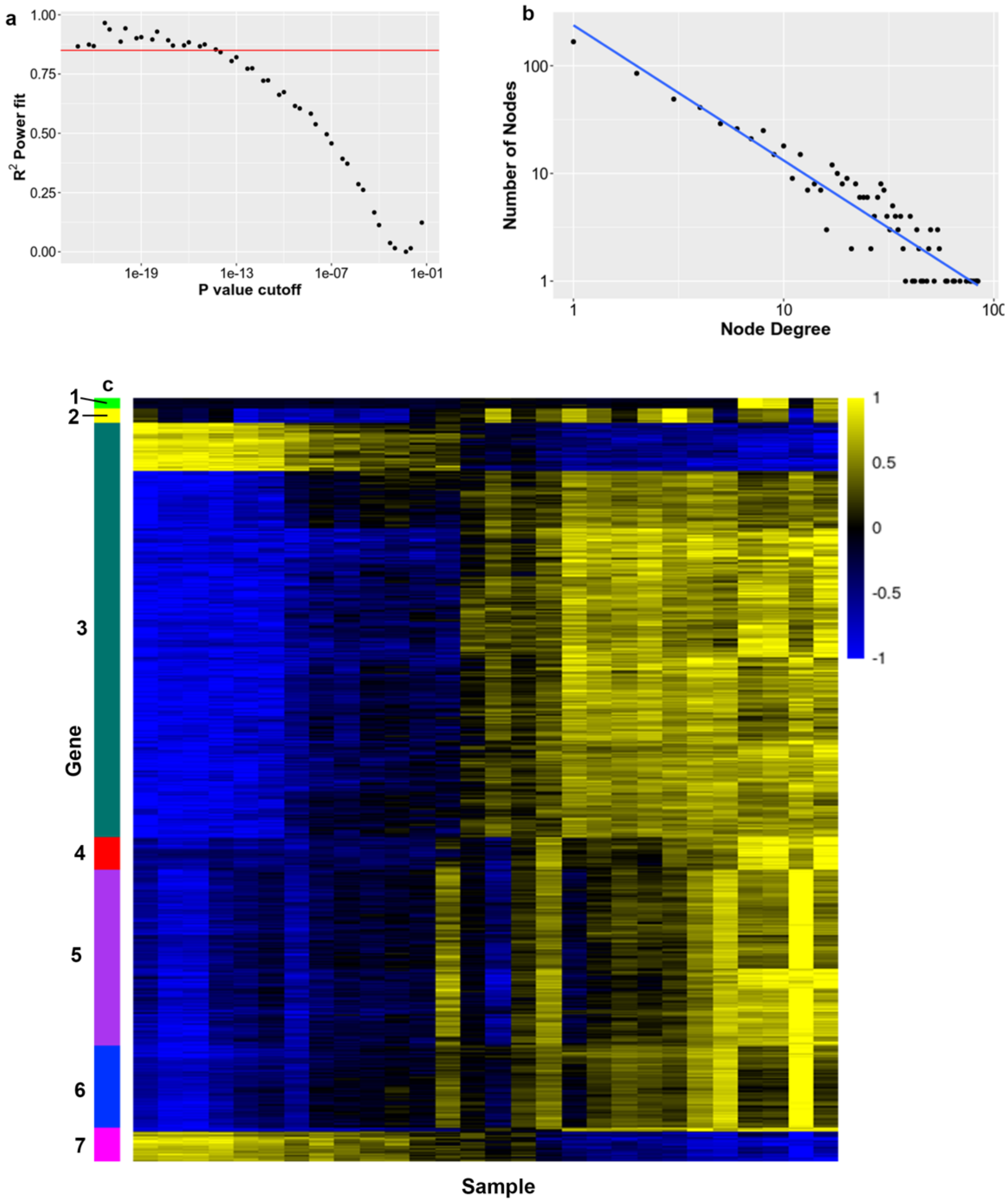

Figure S2: Construction of co-expression network

(a) Plot showing p-value cutoff for significant pairwise pearson correlation coefficients (see Materials and Methods) vs R^2 of a power-fit line. Node degree distributions in biological networks are known to follow a power law, so a p-value cutoff of 1E-16 was chosen, giving an R^2 value of 0.88. **(b)** Plot showing node degree on the x-axis vs the number of nodes with that degree on the y-axis, shown on a log10 scale. A line fits the data with an R^2 value of 0.88, showing that the generated network roughly follows a scale-free distribution. **(c)** Heatmap constructed using SDR values of all genes in all modules. Columns represent samples, ordered by developmental state, and each row represents a gene. Rows were ordered by module, shown as a colored bar on the left. Modules showed distinct expression patterns. The pink4 module was the only module expressed higher during the early embryonic states.
